# Supplementary material for: Sex differences in functional cortical organization reflect differences in network topology rather than cortical morphometry
Source: Nat Commun. 2024 Sep 4;15:7714. doi: 10.1038/s41467-024-51942-1 (PMC11375086; doi:10.1038/s41467-024-51942-1)
Supplement: Supplementary file 1 — Supplementary Information [file 41467_2024_51942_MOESM1_ESM.pdf]

Supplementary Materials for

Sex differences in functional cortical organization reflect differences in network topology rather than cortical morphometry

Bianca Serio*, Meike D. Hettwer, Lisa Wiersch, Giacomo Bignardi, Julia Sacher, Susanne Weis, Simon B. Eickhoff, Sofie L. Valk*

*Correspondence to Bianca Serio (b.serio@fz-juelich.de) and Sofie L. Valk (valk@cbs.mpg.de)

This PDF file includes:

Supplementary Results
Supplementary Figures 1 to 4
Supplementary Tables 1 to 2

Supplementary Methods
Supplementary Table 3
Supplementary Figure 5 to 6
Supplementary Text

Supplementary Results

Figures

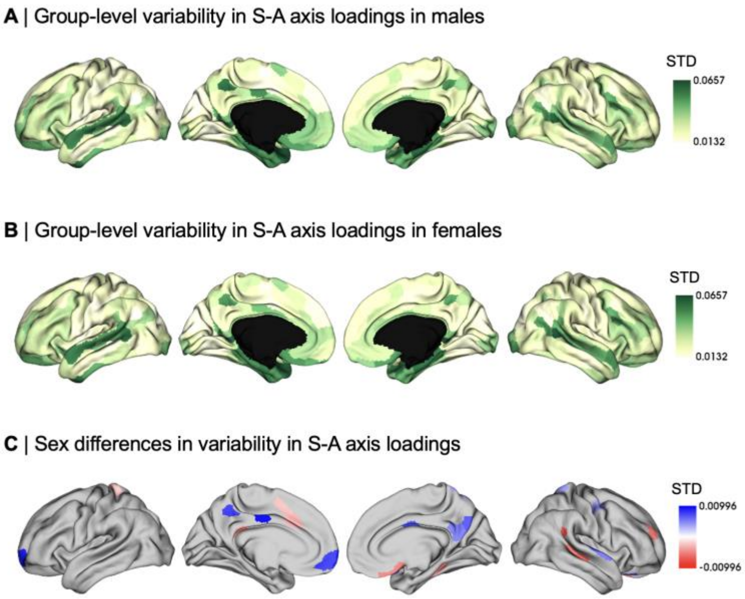

Supplementary Figure 1. Variability in the sensory-association (S-A) axis of functional cortical organization. Group-level variability (quantified by standard deviation; STD) in S-A axis loadings in **A** | males and **B** | females; **C** | Thresholded (false discovery rated (FDR)-corrected, $q < .05$) map of sex differences in variability (standard deviation; STD) as determined by Levene's test for equality of variances, where blue represents higher male variability and red represents higher female variability. Source data are provided as a Source Data file.

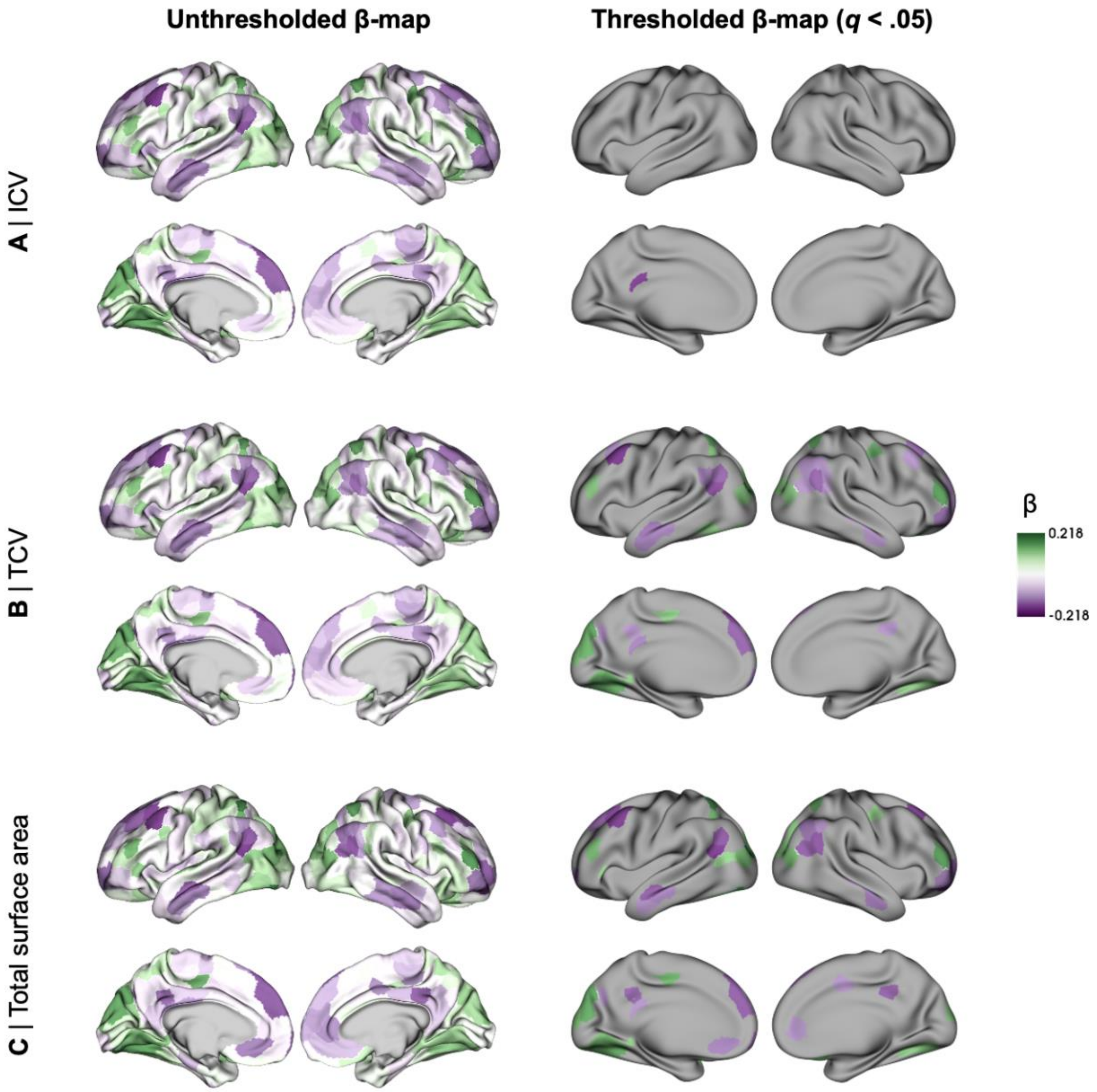

Supplementary Figure 2. Effects of brain size on the sensory-association (S-A) axis of functional cortical organization. Unthresholded and thresholded (false discovery rated (FDR)-corrected, $q < .05$) β-maps of linear mixed effects model results showing the effects of different measures of brain size, namely **A |** intracranial volume (ICV), **B |** total cortical volume (TCV), and **C |** total surface area, on S-A axis loadings. Total surface area yielded the largest number of significant parcels following FDR correction (66), followed by TCV (60), and finally ICV (2). β, standardized beta coefficient. Source data are provided as a Source Data file.

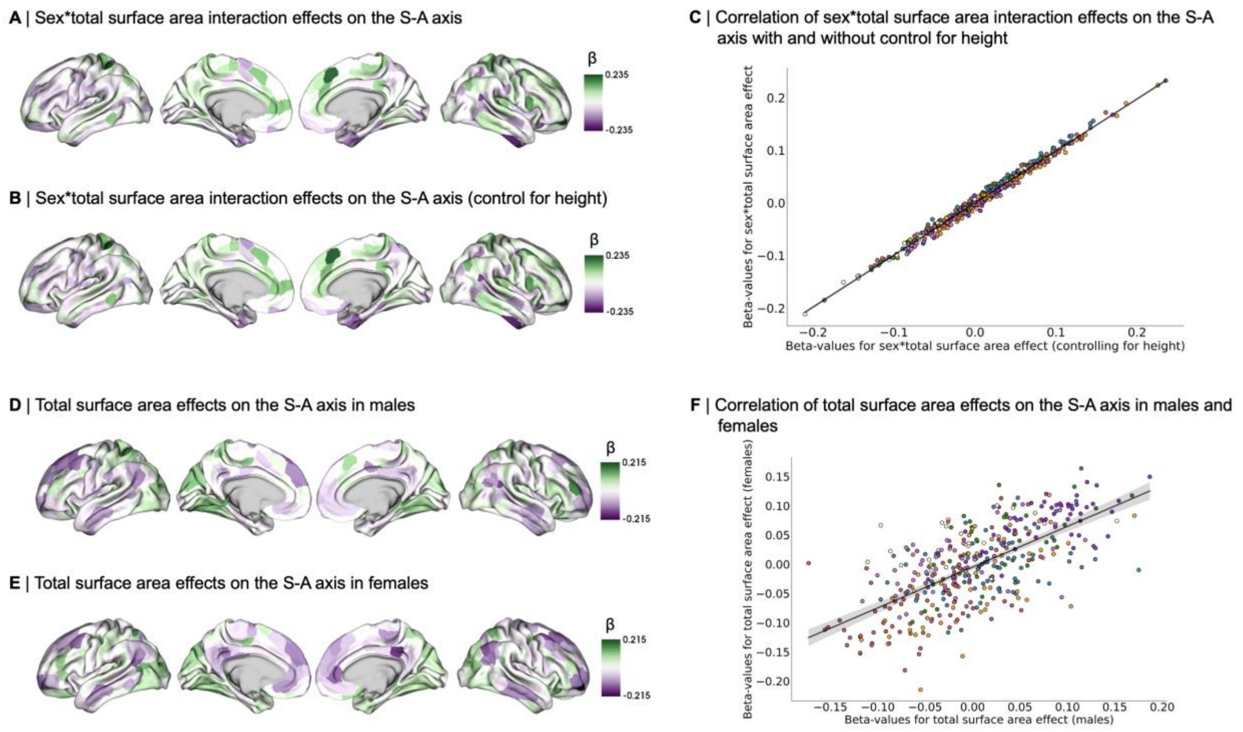

Supplementary Figure 3. Effect of total surface area on the sensory-association (S-A) axis of functional cortical organization and its relationship to sex. A | Unthresholded β-map of linear mixed effect model (LMM) testing for sex by total surface area interaction effect on S-A axis loadings (original result, shown in Figure 4A); **B |** Unthresholded β-map of LMM testing for sex by total surface area interaction effect on S-A axis loadings when including height as a covariate in the LMM; **C |** Scatterplot displaying the spatial correlation between patterns of sex*total surface area interaction effects on S-A axis loadings with (x-axis) and without (y-axis) controlling for height in the LMM (color-coded by Yeo network), tested by a two-sided Spearman correlation and corrected for spatial autocorrelation, r = 0.99, $p_{spin} < .001$; **D |** Unthresholded β-map of LMM testing for sex effect on S-A axis loadings in males; **E |** Unthresholded β-map of LMM testing for sex effect on S-A axis loadings in females; **F |** Scatterplot displaying the spatial correlation between patterns of total surface area effects on S-A axis loadings in males (x-axis) and females (y-axis) (color-coded by Yeo network), tested by a two-sided Spearman correlation and corrected for spatial autocorrelation, r = 0.66, p_{spin} = .002. Error band displays 95% confidence interval. β, standardized beta coefficient. Source data are provided as a Source Data file.

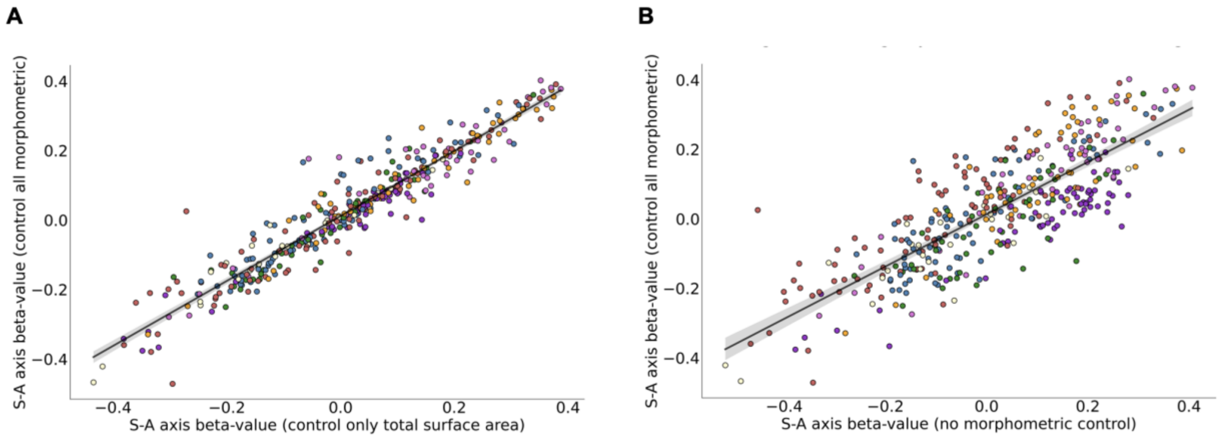

Supplementary Figure 4. Similarity of sex differences in the sensory-association (S-A) axis with and without controlling for morphometric measures. Scatterplots displaying the spatial correlation between patterns of sex effects (β-maps) on S-A axis loadings with (y-axis) and without (x-axis) inclusion of morphometric measures as covariates in the LMMs (color-coded by Yeo network), tested by a two-sided Spearman correlation and corrected for spatial autocorrelation, where **A** | LMM not including morphometric measures (x-axis) still includes total surface area as a covariate (model used to yield main sex difference results, Fig. 1B), $r = 0.95$, $p_{spin} < .001$. Error band displays 95% confidence interval. **B** | LMM not including morphometric measures (x-axis) does not include any morphometric measure as covariates, $r = 0.80$, $p_{spin} < .001$. Error band displays 95% confidence interval. β, standardized beta coefficient. Source data are provided as a Source Data file.

Tables

	Male		Female		β
	**Mean**	**STD**	**Mean**	**STD**	
**ICV (cm^3)**	1'710.35	140.91	1'474.63	149.86	1.25**
**TCV (cm^3)**	1'027.38	87.87	895.34	77.35	1.27**
**Total surface area (cm^2)**	1'947.35	1'542.24	1'715.07	1'469.42	1.21**
**Height (in)**	70.47	2.91	64.91	2.65	1.43**
**Weight (lbs)**	189.93	34.75	156.19	35.82	0.86**
**BMI**	26.84	4.33	26.04	5.66	0.16*

Supplementary Table 1. Sex differences in brain size and anthropometric measurements. Results are yielded by the linear mixed effects model: brain size/anthropometric measurement ~ 1 + sex + age + (1 | family relatedness / sibling status). * indicates $p < 0.05$, ** indicates $p < .001$. STD, standard deviation; β, standardized beta coefficient; ICV, intracranial volume; TCV, total cortical volume; BMI, body mass index.

**WN dispersion (network)**	**β**	***p***	***p*spin**
Visual	-0.251	0.002	0.008
Somatomotor	0.227	0.005	0.013
Dorsal attention	-0.077	0.347	0.094
Ventral attention	0.223	0.007	0.010
Limbic	0.240	0.003	0.023
Fronto parietal	0.192	0.019	0.025
DMN	0.241	0.003	< 0.001*
**BN dispersion (pairwise networks)**	**β**	***p***	***p*spin**
Visual - somatomotor	0.093	0.262	0.338
Visual - dorsal attention	0.044	0.596	0.416
Visual - ventral attention	-0.173	0.038	0.251
Visual - limbic	0.233	0.005	0.167
Visual - fronto parietal	-0.114	0.170	0.343
Visual - DMN	0.073	0.370	0.378
Somatomotor - dorsal attention	-0.046	0.582	0.426
Somatomotor - ventral attention	-0.252	0.003	0.084
Somatomotor - limbic	0.222	0.007	0.210
Somatomotor - fronto parietal	-0.194	0.018	0.172
Somatomotor - DMN	0.007	0.936	0.490
Dorsal attention - ventral attention	-0.251	0.003	0.113
Dorsal attention - limbic	0.213	0.010	0.212
Dorsal attention - fronto parietal	-0.170	0.040	0.232
Dorsal attention - DMN	0.040	0.631	0.425
Ventral attention - limbic	0.311	0.000	0.064
Ventral attention - fronto parietal	0.057	0.494	0.347
Ventral attention - DMN	0.235	0.004	0.081
Limbic - fronto parietal	-0.300	0.000	0.086
Limbic - DMN	-0.212	0.010	0.162
Fronto parietal - DMN	0.240	0.003	0.094

Supplementary Table 2. Sex differences in within-network (WN) and between-network (BN) dispersion. Results are yielded by the linear mixed effects model: dispersion ~ 1 + sex + age + total surface area + (1 | family relatedness / sibling status). * indicates statistical significance at Bonferroni-corrected thresholds of 0.0036 for WN dispersion (7) comparisons, and 0.001 for BN dispersion (21) comparisons. β, standardized beta coefficient; DMN, default mode network.

Supplementary Methods

Race					
**White**	**Black/AM**	**Asian/NH/OPI**	**AI/AN**	**N/A**	**>1**
752 (392)	142 (87)	63 (33)	2 (1)	17 (10)	24 (13)

Ethnicity		
**Not Hispanic/Latino**	**Hispanic/Latino**	**N/A**
896 (491)	91 (39)	13 (6)

Employment		
**Full-time**	**Part-time**	**Not working**
672 (326)	177 (110)	149 (99)

Total household income							
**1**	**2**	**3**	**4**	**5**	**6**	**7**	**8**
69 (43)	72 (32)	132 (60)	118 (67)	99 (56)	207 (115)	139 (78)	157 (81)

Education (years)						
**<11**	**12**	**13**	**14**	**15**	**16**	**17<**
32 (18)	134 (73)	62 (28)	123 (64)	61 (20)	434 (237)	152 (95)

Supplementary Table 3. Sociodemographic breakdown of Human Connectome Project sample. Number of subjects within each category (number of females in parentheses). AM, African American; NH, Native Hawaiian; OPI, Other Pacific Island; AI, American Indian; AN, Alaskan Native; N/A, unknown or not reported; >1, more than one race. Total household income key: 1 = <$10k, 2 = 10k-19'999, 3 = 20k-29'999, 4 = 30k-39'999, 5 = 40k-49'999, 6 = 50k-74'999, 7 = 75k-99'999, 8 = >100k.

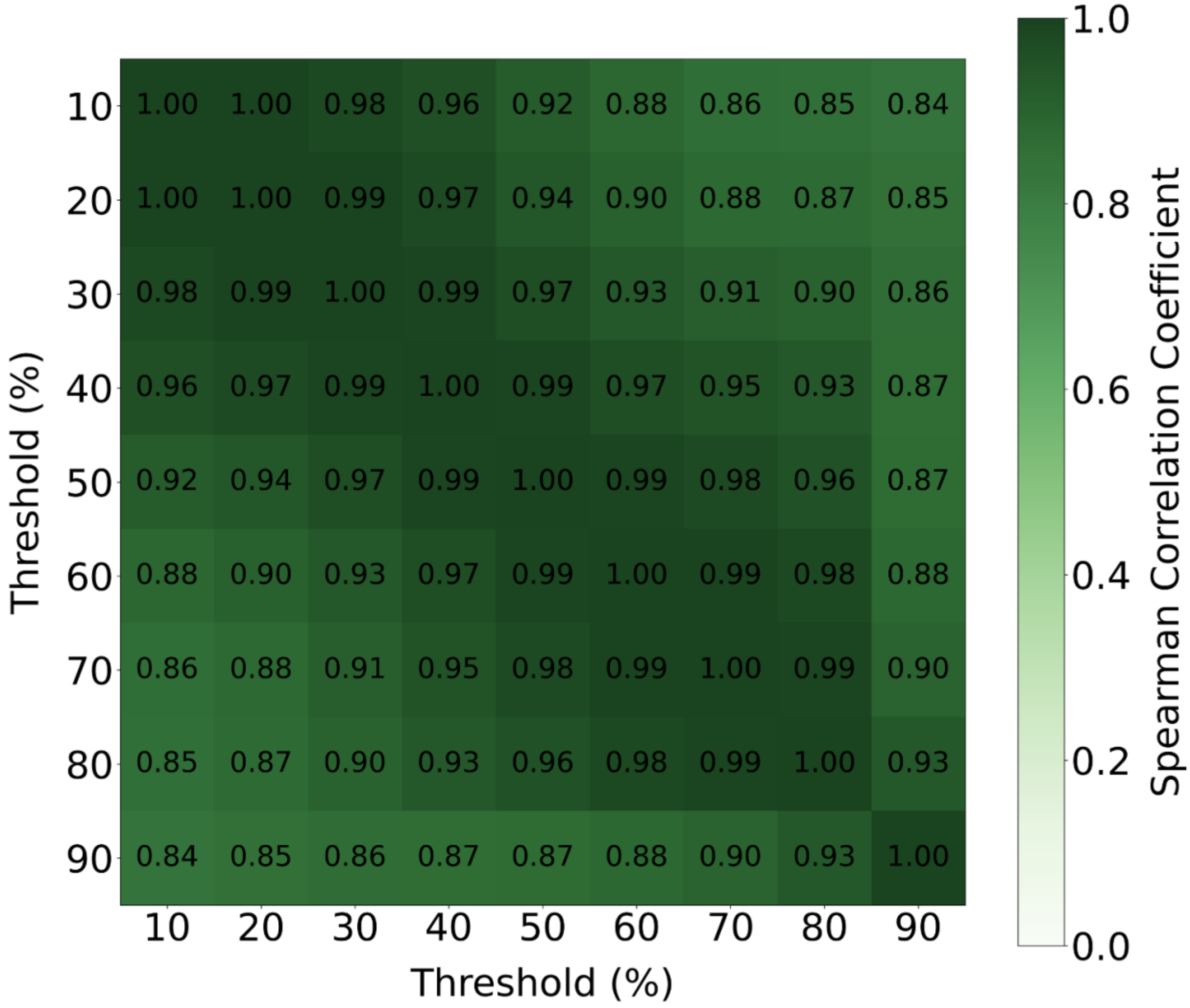

Supplementary Figure 5. Correlation matrix of mean sensory-association (S-A) axis loadings for S-A axes computed using different thresholds.

Concerns have been recently expressed regarding the reliability of the T1w/T2w ratio to quantify microstructural profile intensity (MPI). In fact, MPI is subject to a B1 field bias that has been shown to correlate with demographic and compositional variables such as age, sex, and body mass index (BMI), potentially leading to spurious results when statistical comparing MPI across individuals and groups [77]. However, we did not expect that bias in MPI values would persist in our MPC axis, given that it is an inherently relative measure of intra-individual variation, which is further computed by regressing out mean cortical MPI. Indeed, we show in Supplementary Figure 6 (displayed below) –via correlations between MPI/MPC axis and BMI/ICV– that biases observed when plotting MPI as a function of BMI and ICV (i.e., the distributions of correlation coefficients r(MPI, BMI) (Fig. 6A) and r(MPI, ICV) (Fig. 6C) across sexes are skewed) are not observed when plotting the MPC axis as a function of BMI and ICV (i.e., the distributions of correlation coefficients r(MPC, BMI) (Fig. 6B) and r(MPC, ICV) (Fig. 6D) are normal and do not vary as a function of sex). As such, we confirmed the suitability of MPI, yielded by the T1w/T2w ratio, to compute the MPC axis for further analyses assessing its variation between sexes without introducing bias.

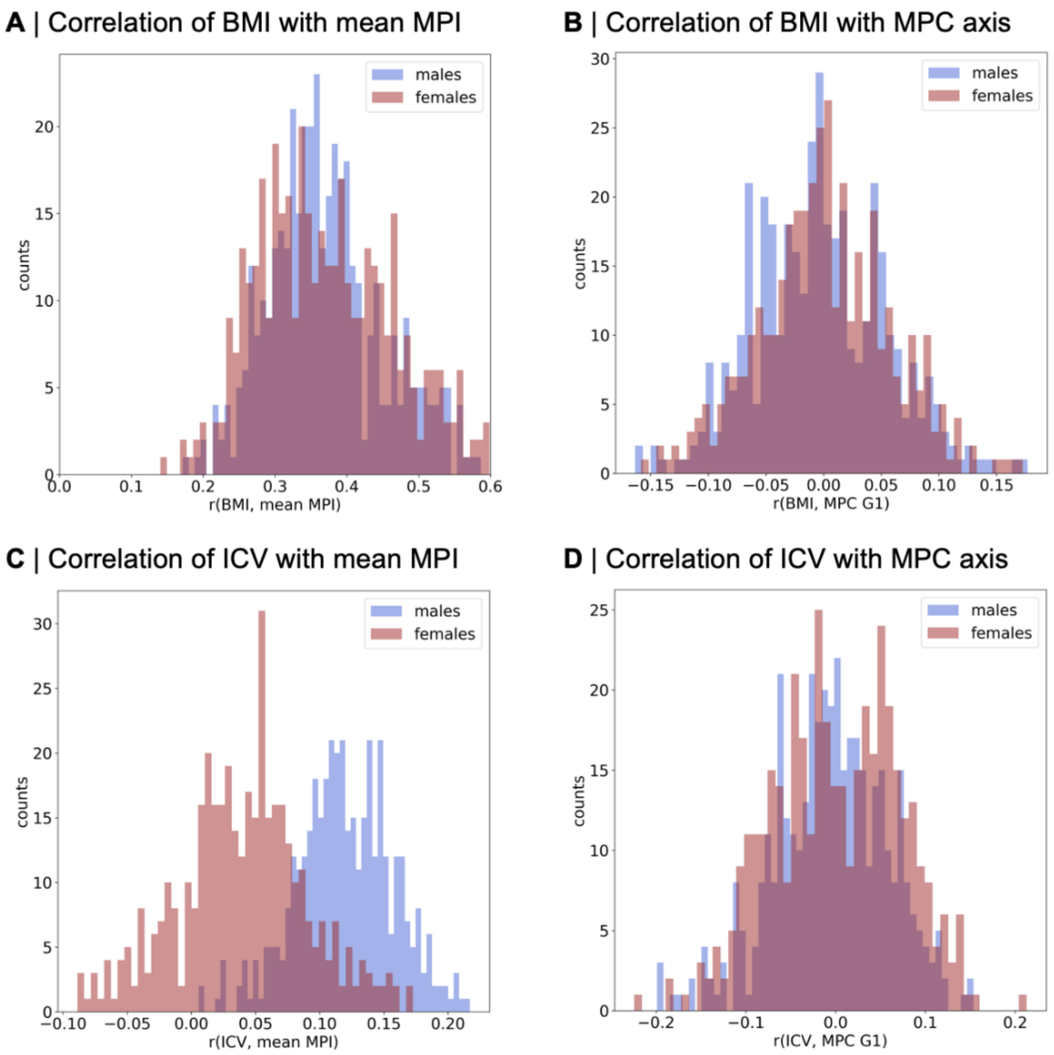

Supplementary Figure 6. Check for field bias in T1w/T2w raw mean microstructure profile intensity (MPI) and derived microstructure profile covariance (MPC) axis. Histograms of correlation coefficients (color-coded by sex) for correlations between: **A** | Body mass index (BMI) and mean MPI (colored by sex); **B** | BMI and mean MPC axis loadings; **C** | Intracranial volume (ICV) and mean MPI (colored by sex); **D** | ICV and mean MPC axis loadings. Source data are provided as a Source Data file.
