# Supplementary material for: Sex differences in functional cortical organization reflect differences in network topology rather than cortical morphometry
Source: Nat Commun. 2024 Sep 4;15:7714. doi: 10.1038/s41467-024-51942-1 (PMC11375086; doi:10.1038/s41467-024-51942-1)
Supplement: Supplementary file 3 — Reporting Summary [file 41467_2024_51942_MOESM3_ESM.pdf]

nature portfolio

Corresponding author(s): Bianca Serio, Dr. Sofie L. Valk

Last updated by author(s): Jul 20, 2024

Reporting Summary

Nature Portfolio wishes to improve the reproducibility of the work that we publish. This form provides structure for consistency and transparency in reporting. For further information on Nature Portfolio policies, see our Editorial Policies and the Editorial Policy Checklist.

Statistics

For all statistical analyses, confirm that the following items are present in the figure legend, table legend, main text, or Methods section.

n/a	Confirmed	
☐	☒	The exact sample size (n) for each experimental group/condition, given as a discrete number and unit of measurement
☐	☒	A statement on whether measurements were taken from distinct samples or whether the same sample was measured repeatedly
☐	☒	The statistical test(s) used AND whether they are one- or two-sided<br>*Only common tests should be described solely by name; describe more complex techniques in the Methods section.*
☐	☒	A description of all covariates tested
☐	☒	A description of any assumptions or corrections, such as tests of normality and adjustment for multiple comparisons
☐	☒	A full description of the statistical parameters including central tendency (e.g. means) or other basic estimates (e.g. regression coefficient) AND variation (e.g. standard deviation) or associated estimates of uncertainty (e.g. confidence intervals)
☐	☒	For null hypothesis testing, the test statistic (e.g. F, t, r) with confidence intervals, effect sizes, degrees of freedom and P value noted<br>*Give P values as exact values whenever suitable.*
☒	☐	For Bayesian analysis, information on the choice of priors and Markov chain Monte Carlo settings
☒	☐	For hierarchical and complex designs, identification of the appropriate level for tests and full reporting of outcomes
☐	☒	Estimates of effect sizes (e.g. Cohen's d, Pearson's r), indicating how they were calculated

Our web collection on statistics for biologists contains articles on many of the points above.

Software and code

Policy information about availability of computer code

Data collection	We obtained human data from the open-access Human Connectome Project HCP S1200 young adult sample. Data are freely available at http://www.humanconnectome.org/.
Data analysis	Analyses were conducted in Python and R: The code used in this manuscript is available at https://github.com/biancaserio/sex_diff_gradients (v1; https://zenodo.org/doi/10.5281/zenodo.12785462). The code and tutorials for functional gradient decomposition and to generate geodesic distances can further be found at https://brainspace.readthedocs.io/en/latest/index.html and https://micapipe.readthedocs.io/en/latest/ respectively.

For manuscripts utilizing custom algorithms or software that are central to the research but not yet described in published literature, software must be made available to editors and reviewers. We strongly encourage code deposition in a community repository (e.g. GitHub). See the Nature Portfolio guidelines for submitting code & software for further information.

Data

Policy information about availability of data

All manuscripts must include a data availability statement. This statement should provide the following information, where applicable:

- Accession codes, unique identifiers, or web links for publicly available datasets
- A description of any restrictions on data availability
- For clinical datasets or third party data, please ensure that the statement adheres to our policy

We obtained human data from the open-access Human Connectome Project HCP S1200 young adult sample. Data are freely available at http://

www.humanconnectome.org/.

Research involving human participants, their data, or biological material

Policy information about studies with human participants or human data. See also policy information about sex, gender (identity/presentation), and sexual orientation and race, ethnicity and racism.

Reporting on sex and gender	Our study primary aim is to investigate sex differences. We refer to sex as a biological variable (self-reported biological sex as opposed to gender identification). We intentionally did not consider gender in our study as our research aims specifically focus on identifying biological and anatomical mechanisms relating to associations between cortical morphometry and functional organization. We do however mention in our study limitations that -despite being beyond the scope of our study- we neglected possible effects of gender, and that findings may appear more nuanced if we moved beyond the assumption of a clear-cut sexual dimorphism of brain structure and function, as the relevance of considering transgender individuals in the study of sex differences is being increasingly recognized.
Reporting on race, ethnicity, or other socially relevant groupings	Our study sample is from the Human Connectome Project. Subjects were all born in Missouri but recruited in an attempt to broadly reflect the racial and ethnic composition of the United States population. We did not control for confounding variables of race, ethnicity or socially relevant groupings as these groupings may further reflect further social-environmental variables whose variance we did not want to remove from our models focusing on biological associations between cortical morphometry and functional organization.
Population characteristics	Our analyses were conducted on the publicly available data of healthy young adults from the Human Connectome Project (HCP) S1200 release, with a sample of 1000 individuals (536 females) with a mean age of 28.73 ± 3.71 years, including 284 monozygotic twins (MZ), 184 dizygotic twins (DZ), 443 non-twin siblings, and 89 unrelated individuals. The term "healthy" was thus broadly defined. Individuals with documented neurodevelopmental and psychiatric disorders, or reporting physiological illnesses such as high blood pressure or diabetes were excluded, but not individuals who reported smoking, being overweight, or a history of recreational drug use or heavy drinking (if they had not experienced severe symptoms). Informed consent was obtained for all study subjects.
Recruitment	Recruitment efforts aimed to yield a subject pool capturing a wide range of variability –in socioeconomic and behavioral terms– in order to be representative of the general healthy population. Subjects were all born in Missouri but recruited in an attempt to broadly reflect the racial and ethnic composition of the United States population, as well as belonging to families that include twins based on data from the Missouri Department of Health and Senior Services Bureau of Vital Records. Identified prospective subjects then underwent telephone screening to ascertain whether they meet the HCP inclusion criteria.
Ethics oversight	Washington University, University of Minnesota, and the Oxford University Consortium

Note that full information on the approval of the study protocol must also be provided in the manuscript.

Field-specific reporting

Please select the one below that is the best fit for your research. If you are not sure, read the appropriate sections before making your selection.

[x] Life sciences [ ] Behavioural & social sciences [ ] Ecological, evolutionary & environmental sciences

For a reference copy of the document with all sections, see nature.com/documents/nr-reporting-summary-flat.pdf

Life sciences study design

All studies must disclose on these points even when the disclosure is negative.

Sample size	The Human Connectome Project consortium undertook a systematic effort to macroscopically map the human brain in a large population of healthy adults - collecting 1200 subjects in total.<br>We have not originally conducted a power analysis to inform our sample size given that the HCP sample of N = 1000 conforms to suggestions of sample sizes and power required in brain-wide association studies (see Marek et al. (2022) Nature). In fact, we did find effects of both sex and morphometric measures on the S-A axis, as well as sex effects on morphometric measures, suggesting that power was appropriate, at least for detecting sex effects.
Data exclusions	We selected subjects with available functional, T1, and T2 data, resulting in a final sample of 1000 individuals.
Replication	We did not replicate our current findings in another sample due to the unique multi-modal nature of our current work.
Randomization	Randomization is not relevant for our study given that there are no experimental groups. We allocated males and females to their respective self-reported biological sex.
Blinding	Blinding was not relevant to this study as there was no experiment per se that required blinding. Data was either self-reported by subjects or collected in a magnetic resonance imaging scanner.

Reporting for specific materials, systems and methods

We require information from authors about some types of materials, experimental systems and methods used in many studies. Here, indicate whether each material, system or method listed is relevant to your study. If you are not sure if a list item applies to your research, read the appropriate section before selecting a response.

Materials & experimental systems

n/a	Involved in the study
☒	☐ Antibodies
☒	☐ Eukaryotic cell lines
☒	☐ Palaeontology and archaeology
☒	☐ Animals and other organisms
☒	☐ Clinical data
☒	☐ Dual use research of concern
☒	☐ Plants

Methods

n/a	Involved in the study
☒	☐ ChIP-seq
☒	☐ Flow cytometry
☐	☒ MRI-based neuroimaging

Plants

Seed stocks: n/a

Novel plant genotypes: n/a

Authentication: n/a

Magnetic resonance imaging

Experimental design

Design type: Resting state

Design specifications: No blocks/trials in resting state design. A total of 1h of resting-state functional data was collected across four identical 15min scanning sessions, equally split over two days.

Behavioral performance measures: No behavioral or task measures recorded in resting state design.

Acquisition

Imaging type(s): Structural and functional

Field strength: 3T

Sequence & imaging parameters: Structural MRI images were acquired via high resolution T1-weighted (T1w) and T2-weighted (T2w) sequences. Two separate T1w images were acquired and averaged, with identical scanning parameters using a 3D MPRAGE sequence (0.7 mm isovoxels, FOV = 224 mm, matrix=320×320 mm, 256 sagittal slices; TR=2400 ms, TE=2.14 ms, TI=1000 ms, flip angle=8°, BW = 210 Hz per pixel, ES = 7.6 ms). Two separate T2w images were acquired and averaged, with identical scanning parameters using a variable flip angle turbo spin-echo (3D T2-SPACE) sequence, with the same isotropic resolution, matrix, FOV, and slices as for the T1w sequence (TR=3200 ms, TE=565 ms, BW = 744 Hz per pixel, total turbo factor = 314).

The functional resting state data was collected with a gradient echo EPI sequence at a resolution of 2 mm isotropic (FOV = 208 × 180 mm, matrix = 104 × 90 mm, 72 slices covering the whole brain, TR = 720 ms, TE = 33 ms, multiband factor of 8, FA = 52°).

Area of acquisition: Whole brain

Diffusion MRI: ☐ Used ☒ Not used

Preprocessing

Preprocessing software	The HCP structural pipelines use FreeSurfer 5.1 software plus a series of customized steps that combine information from both T1w and T2w scans for more accurate white and pial surfaces.
Normalization	The multimodal surface matching algorithm (MSMAll) was used to co-register the data to the HCP template 32 k_LR surface space.
Normalization template	The HCP template 32 k_LR surface space consists of 32492 nodes per hemisphere (59412 nodes excluding the medial wall).
Noise and artifact removal	Independent component analysis (ICA) was applied to each 15-minute rfMRI dataset to remove structured artifacts from the minimally preprocessed data. FSL's MELODIC tool was used to decompose the data into multiple (typically ~ 230) components, each comprising a single spatial map and an associated timecourse. Some components represent artifacts such as head motion or cardiac pulsation, while others represent valid neuronally-related spontaneous fluctuations. A tool called 'FIX' (FMRIB's ICA-based X-noiseifier) was used to automatically classify components into "bad" versus "good". The bad components' timeseries were then regressed out of the data, along with various head-motion-related confound regressors.
Volume censoring	We did not perform volume censoring.

Statistical modeling & inference

Model type and settings	We used vertex-wise functional timeseries -averaged within the Schaefer 400 cortical parcels- for our analyses.
Effect(s) tested	No task/stimulus in resting state design.

Specify type of analysis: ☒ Whole brain ☐ ROI-based ☐ Both

Statistic type for inference (See Eklund et al. 2016)	Not applicable in resting state design.
Correction	We used FDR correction to correct for multiple comparisons across the 400 Schaefer parcels.

Models & analysis

n/a	Involved in the study
☐	☒ Functional and/or effective connectivity
☒	☐ Graph analysis
☒	☐ Multivariate modeling or predictive analysis

Functional and/or effective connectivity	FC matrices (400x400) were computed at the individual level –per scanning session– by correlating cortical timeseries in a pairwise manner using the Pearson product moment. We normalized the correlation coefficients using Fisher's z-transformation. Final FC matrices were obtained by averaging each subject's matrices across their four scanning sessions.
